# Mutational Analysis of Photosystem I of *Synechocystis* sp. PCC 6803: The Role of Four Conserved Aromatic Residues in the *j*-helix of PsaB

**DOI:** 10.1371/journal.pone.0024625

**Published:** 2011-09-12

**Authors:** Wu Xu, Yingchun Wang, Eric Taylor, Amelie Laujac, Liyan Gao, Sergei Savikhin, Parag R. Chitnis

**Affiliations:** 1 Department of Chemistry, University of Louisiana at Lafayette, Lafayette, Louisiana, United States of America; 2 Key Laboratory of Molecular and Developmental Biology, Institute of Genetics and Developmental Biology, Chinese Academy of Science, Beijing, China; 3 Department of Physics, Purdue University, West Lafayette, Indiana, United States of America; 4 Department of Biochemistry, Biophysics and Molecular Biology, Iowa State University, Ames, Iowa, United States of America; Auburn University, United States of America

## Abstract

Photosystem I is the light-driven plastocyanin-ferredoxin oxidoreductase in the photosynthetic electron transfer of cyanobacteria and plants. Two histidyl residues in the symmetric transmembrane helices A*-j* and B*-j* provide ligands for the P700 chlorophyll molecules of the reaction center of photosystem I. To determine the role of conserved aromatic residues adjacent to the histidyl molecule in the helix of B-*j*, we generated six site-directed mutants of the *psaB* gene in *Synechocystis* sp. PCC 6803. Three mutant strains with W645C, W643C/A644I and S641C/V642I substitutions could grow photoautotrophically and showed no obvious reduction in the photosystem I activity. Kinetics of P700 re-reduction by plastocyanin remained unaltered in these mutants. In contrast, the strains with H651C/L652M, F649C/G650I and F647C substitutions could not grow under photoautotrophic conditions because those mutants had low photosystem I activity, possibly due to low levels of proteins. A procedure to select spontaneous revertants from the mutants that are incapable to photoautotrophic growth resulted in three revertants that were used in this study. The molecular analysis of the spontaneous revertants suggested that an aromatic residue at F647 and a small residue at G650 may be necessary for maintaining the structural integrity of photosystem I. The (P700^+^ - P700) steady-state absorption difference spectrum of the revertant F647Y has a ∼5 nm narrower peak than the recovered wild-type, suggesting that additional hydroxyl group of this revertant may participate in the interaction with the special pair while the photosystem I complexes of the F649C/G650T and H651Q mutants closely resemble the wild-type spectrum. The results presented here demonstrate that the highly conserved residues W645, W643 and F649 are not critical for maintaining the integrity and in mediating electron transport from plastocyanin to photosystem I. Our data suggest that an aromatic residue is required at position of 647 for structural integrity and/or function of photosystem I.

## Introduction

Photosystem I (PS I) is a protein-pigment complex in cyanobacteria and higher plants. It mediates the light-driven electron transfer from plastocyanin to ferredoxin [1]. High resolution structures for cyanobacterial [2] as well as for higher plant PS I [3], [4] are known from X-ray crystallographic studies. Monomers of cyanobacterial PS I complex contain twelve protein subunits, ninety six chlorophyll a molecules, twenty two β-carotenes, two phylloquinones, four lipid molecules, and three [4Fe-4S] clusters. Structure of cyanobacterial PS I is known at 2.5 Å resolution and the available tools of reverse genetic study for cyanobacterial PS I make it an excellent system to investigate the role of protein environment in modulating spectral, redox, and electron transfer properties of cofactors in a complex system [2], [5]. The PsaA and PsaB proteins of PS I form the core that binds the P700, which is a dimer of chlorophyll a and a′ molecules, and the chain of electron acceptors A0 (a chlorophyll a molecule), A1 (a phylloquinone) and FX (a [4Fe-4S] cluster). In addition, the core binds most other cofactors. The peripheral PsaC subunit binds the terminal electron acceptors, FA and FB, both of which are [4Fe-4S] clusters. Upon receiving energy of a photon, excitation of P700 leads to charge separation. An electron is transferred to A0, then to A1 and ultimately to a ferredoxin molecule through the series of three [4Fe-4S] clusters [6], [7]. On the lumenal side of thylakoid membranes, the PS I complex accepts electrons from plastocyanin. In cyanobacteria and algae, cytochrome c6 can functionally replace plastocyanin-deficient strains [8] as well as under copper-deficient conditions [9]. However, Arabidopsis plants mutated in both plastocyanin-coding genes and with a functional cytochrome c6 cannot grow photoautotrophically because of a complete blockade in light-driven electron transport, demonstrating that in Arabidopsis only plastocyanin can donate electrons to photosystem I in vivo [10].

Protein environment around the reaction center P700 has many major functions: to maintain structure around the reaction center, to impart special properties of the P700 chlorophyll pair, and to provide pathway(s) for transfer of electrons from the docked plastocyanin (or cytochrome *c*_6_) to P700. In the 2.5 Å resolution crystal structure of PS I, two chlorophyll molecules of P700 are confined by symmetrically positioned four α-helices, A-*j*, A*-k* and B*-j*, B*-k* [2]. The histidyl residues in A*-j* and B*-j* α-helices provide the fifth ligands to both Mg^2+^ of the chlorophyll dimer of P700 [11], [12]. Depending on an organism, the electron donor molecules to PS I dock on specific sites on the lumenal side of PS I [13]–[16]. Specific residues in PsaF form integral components of the docking site [16]. Additionally, plastocyanin or cytochrome *c*_6_ interacts directly with the PsaA and PsaB core subunits [15], [17], [18] and donate electrons to P700^+^ [17], [19]–[22]. The kinetic data of PS I reduction by plastocyanin corresponds to a monophasic process while the PS I reduction by cytochrome *c*_6_ follows biphasic kinetics with the first fast component in the microsecond range [13], [22]. This fast phase of PS I reduction has been typically described by a kinetic model involving transient complex formation before the electron-transfer step [13], [22], suggesting that cytochrome *c*_6_ interacts with PS I and donates an electron to P700^+^ *in vivo* following a mechanism more complex and more efficient than that of plastocyanin although there are discrepancies between *in vivo* and *in vitro* results [23]. Since P700 is positioned 10–15 Å away from lumenal surface [24], the protein components filling the space between the electron donor and P700 may provide a pathway for directly or indirectly migrating electrons that reduce P700^+^.

**Figure 1:**
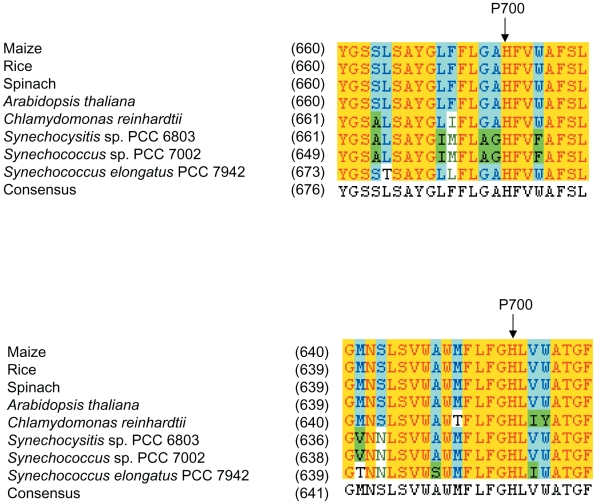
Amino acid sequence alignments of the A*-j and* B*-j* helices of the PS I core. The deduced protein sequences of the *j*-helices from the PsaA and PsaB proteins of maize (*Zea mays*), rice (*Oryza sativa*), spinach (*Spinacia oleracea*), *Arabidopsis thaliana*, *Chlamydomonas reinhardtii*, *Synechocystis* sp. PCC 6803, *Synechococcus* sp. PCC 7002 and *Synechococcus elongatus* PCC 7942 were aligned using Invitrogen software (Carlsbad, CA). P700 ligands are indicated (P700).

In an attempt to dissect the special structural properties of the P700 environment in the PsaB side, we performed site-directed mutagenesis of residues near P700. A major feature of the helical regions near P700 is the presence of several aromatic residues. When we compared primary sequences of the A*-j* and B*-j* helices, the *j* helix of PsaB was found to contain four highly conserved aromatic amino acid residues, W643, W645, F647 and F649 (Figure 1). This arrangement of aromatic residues is absent in the corresponding region of PsaA (Figure S1). We postulated that one or more of these aromatic residues in PsaB could be involved in maintaining the architecture around P700. It is also possible that these aromatic residues are integral components of the electron transfer path between the redox centers of plastocyanin or cytochrome *c*_6_ and P700^+^ since it was reported that side chains of aromatic residues can serve as an electron tunneling bridge [25], [26]. One recent report indicated that photo-oxidation of the chlorophyll *a/a*′ heterodimer, P700, causes shifts in the vibrational frequencies of two or more tryptophan residues in PS I, demonstrating that role of aromatic residues in electron transfer [27]. To test these hypotheses, we replaced each of the four aromatic residues with cysteinyl residues. Two additional conserved residues in the B-*j* helix, H651 and S641, were also replaced by cysteinyl residues. H651 provides the fifth ligand to Mg^2+^ of P700 on PsaB side. Here we present properties of the mutant strains and their PS I complexes.

## Materials and Methods

### Cyanobacterial culture

Cells of *Synechocystis* sp. PCC 6803 [17] (thereafter *Synechocystis*) were cultured at 30°C in BG-11 medium [17], [28], [29] supplemented with glucose (5 mM) and appropriate antibiotics (30 mg/L chloramphenicol or 50 mg/L kanamycin). Liquid cultures were aerated by shaking at 120 rpm or by bubbling with air. Different light intensities were used depending on the strains or experiments: low (2–3 µmoles m^−2^ s^−1^), medium (40 µmoles m^−2^ s^−1^), and high (160 µmoles m^−2^ s^−1^). The growth of *Synechocystis* cells was monitored from density of cell cultures measured as absorbance at 730 nm (A_730 nm_), which was determined using an UV-160U spectrophotometer. Cells were harvested during the exponential growth phase, resuspended in 0.4 M sucrose, 10 mM NaCl, 10 mM MOPS-HCl (pH 7.0), and stored at −20°C for further use [17].

### Plasmids and site-directed mutagenesis

Plasmid pGEM3C+ contains the C-terminal region of the *psaB* gene, resistance genes for chloramphenicol and ampicillin, and 760 bp region down stream of the *psaB* gene [30], [31]. A PCR-based method [32] was used to generate the mutant recombinant DNAs using pGEM3C+ as a template. Table S1 lists the oligonucleotides that were used to engineer the mutant DNAs. To clone the amplified DNAs, the fragments, the third PCR products were digested with *Eag* I and *Apa* I and ligated into pGEM3C+ that had been digested with the same enzymes. The amplified regions from the ligated pGEM3C+ were sequenced completely to confirm the presence of desired mutations and to ensure fidelity of *Taq* polymerase.

### Transformation of Synechocystis sp. PCC 6803

Plasmid DNAs with appropriate recombinant constructions were used to transform the recipient strain PCRTΔB. Transformation and selection of transformants were performed under low light intensity (2–3 µmoles m^−2^ s^−1^) at 30°C according to the references [29]–[31]. Chloramphenicol-resistant transformants were selected, segregated for more than three generations and replica-plated to confirm the absence of kanamycin-resistance gene. After segregation, the genomic DNAs were isolated from the mutant strains. The fragments containing the mutated sites were amplified by PCR and the fragments were sequenced to confirm the mutations. The pGEM3C+ plasmid with wild type gene was introduced back into the recipient strain PCRTΔB to generate the recovered wild type (RWT) which serves as a positive control for these studies.

### Physiological characterization of the mutant strains

All mutants, recipient strain and RWT were cultured in the BG-11 medium with 5 mM glucose under 2–3 µmoles m^−2^ s^−1^ light and were harvested at approximately 0.8 A_730 nm_/ml of the culture. Cells were centrifuged at 4,000 g and pellet was resuspended in BG-11 medium. The cells were pelletized again and the procedure was repeated three times to remove all glucose. Cells were grown at 30°C with or without glucose. Cultures were shaken constantly at 120 rpm under different light intensities.

### Biochemical characterization of the mutant strains

Isolation of thylakoid membranes and measurement of chlorophyll content were performed according previously published methods [33]. Light-driven PS I-mediated electron transport from DAD/PC to MV was monitored using a Clark type oxygen electrode (Hansatech, England) and used to examine electron transport activity of PS I [34], [35]. The PS I activity in the membranes was also determined with NADP^+^ photoreduction assay using cytochrome *c*_6_ and ferredoxin as electron donor and acceptor, respectively [36]. PS II activity was measured as the light-driven oxygen evolution, in which electrons are transferred from water to *p*-benzoquinone *via* the PS II complex [37]. Accumulation of PS I proteins was examined by analytical SDS-PAGE and immunodetection [38]. The antibodies for detection of PS I proteins have been described previously [36]. The PS I complexes were purified and the mutant PS I complexes were modified by biotin-maleimide according to a previously published method [36]. For transient absorption spectroscopy, plastocyanin from *Synechocystis* sp. PCC 6803 was purified as described previously [39]. Kinetics of flash-induced absorbance changes in PS I was followed at 820 nm. The experimental set-up, data collection and kinetic analyses have been described previously [13]. For experiments at varying ionic strength, the magnesium salt was omitted and the ionic strength was adjusted at the desired value by adding NaCl.

### Generation and selection of spontaneous revertants

The mutant strains, that require glucose for their growth, were cultured with 1 mM glucose under normal light and with air in one-liter bottle for ten days. Glucose concentration for screening revertants is five times less than the normal concentration in order to increase the mutational frequency. The surviving cells were collected and spread on the nutrient plates without glucose. The plates were incubated under 40 µmoles m^−2^ s^−1^ light at 30°C to screen for revertant colonies. This process was repeated until single colonies appeared in plates after approximate 40 days. The revertant colonies were grown in liquid medium for further analysis. The genomic DNAs were isolated from each revertant. The fragments containing the mutated sites were amplified by PCR and the fragments were sequenced to examine the molecular nature of reversion.

### Spectroscopic characterizations of the revertants

The optical pump-probe spectroscopy system has been described in detail elsewhere [40], [41]. Pulses from a self-mode-locked Ti:sapphire laser were amplified by a factor of ∼10^5^ at 1 kHz repetition rate in a regenerative amplifier. The 780 nm output was converted to infrared signal and idler pulses in a Type I BBO optical parametric amplifier. The signal output pulses were frequency-doubled into tunable visible light pulses (600–730 nm), which served as sample excitation pulses. Sample absorption was probed with single filament broadband continuum light pulses generated in a sapphire plate; cross-correlations between the pump and probe pulses were typically 100–200 fs fwhm. Continuum pulses were split into signal and reference beams, dispersed in an Oriel MS257 imaging monochromator operated at ∼3 nm bandpass, and directed onto separate Hamamatsu S3071 Si pin photodiodes. Noise performance was near shot noise-limited; the rms noise in Δ*A* was ∼10^−5^ for 1 sec accumulation time. Operation in the annihilation-free regime was ensured by control experiments in which the pump power was varied; all experiments were performed at room temperature. Unless otherwise specified, the pump and probe polarizations were separated by 54.7° resulting in isotropic kinetic measurements.

All PS I samples contained 20 mM sodium ascorbate. As shown by Savikhin et al. [42], samples in experiments conducted in total darkness contained predominantly open reaction centers; continuous illumination of the sample cell by a 3 V flashlight bulb yielded samples in which the reaction centers were almost exclusively closed. While it is known that recombination occurs from the terminal acceptor (F_A/B_) with ∼45 ms kinetics [43], we have found that essentially all of the reaction centers are converted into P700^+^ after ∼4 sec of continuous illumination; regeneration of P700 under dark conditions requires ∼120 sec in 20 mM ascorbate solution for WT. The latter process occurs directly from the ascorbate anion: its kinetics are single-exponential, with rate constant proportional to ascorbate concentration for concentrations ≥20 mM. 1–5% of the unpaired electrons on F_A/B_^−^ are typically scavenged from the PS I complex prior to recombination, and each PS I complex is excited hundreds of times during 4 sec illumination. Hence, while continuous illumination can cause substantial population buildup of long-lived P700^+^, such buildup is negligible under short-pulse excitation [43]. Steady-state absorption spectra of PS I samples with open and closed reaction centers were accumulated in a Perkin-Elmer Lambda 3B spectrophotometer. The spectrophotometer modification for low-noise measurement of absorbance changes in samples with closed and open reaction centers has been described earlier [42].

### Theoretical study of the point mutations

We performed a computational modeling study of a decapeptide fragment of PS I. The study focused on a target decapeptide of PsaB from *Synechococcus elongates* (PDB ID: 1JB0), amino acids 650–661 and P700 (Chlorophyll ID: A1011 and B1021). For inclusion of amino acid environment, this study considered only PsaA and PsaB, some 23,000+ atoms in all. This entailed removal of all other polypeptide chains, chlorophylls, iron sulfur moieties, waters of hydration and other noncovalent species associated with either PsaA or PsaB. Amino acid replacements to derive the mutant cases followed the replacements in the experimental protocols previously discussed and nomenclatures were correspondingly changed to *Synechocystis* in order to easily correlate this theoretical study to the experimental results. In examining the mutational effects of replacing amino acids from an energy standpoint, it was necessary to retain the molecular environment of the targeted amino acids. Thus examination of the energy of the native amino acid sequences and then the energy of the mutant versions allowed direct comparison within the same overall molecular environment of PsaA and PsaB. The energy determinations involved first establishing a base-line energy of the overall the WT PsaA and PsaB. Replacement of each targeted amino acid with its mutant amino acid then permitted determination of the energy of each mutant system in turn.

The computational study employed HyperChem 7.52 (Hypercube, Inc., Gainesville, FL, USA). In examining the WT structure with HyperChem, the chlorophylls did not exhibit any atomic charges. We employed the Extended Hückel method embodied within the HyperChem program for assigning atomic charges to the chlorophyll moieties. We used Mm+ of the HyperChem program as the method for determining energies. Utilizing the HyperChem Single Point (SP) energy calculation method, we established a base-line energy, E_WT_, of the WT PsaA and PsaB. The SP energy offers static properties of the target molecule. The energy includes potential energy, electrostatic potential, molecular orbital energies, *etc*. By repeating this SP calculation for each minimized mutant variant in turn, each yielded an energy, E_mut_, permitting the determination of each mutant's ΔE_mut_ = E_mut_−E_WT_. Since replacing a native amino acid with another, the mutant, the mutant amino acid side chain would not likely be minimized as to its environment energy within PsaA and PsaB as initially placed in the PS I fragment, though the native peptide conformation was retained for the mutant. Before determining ΔE_mut_, each mutant case amino acid replacement was minimized within its local environment using Mm+. Once minimized, the SP energy was determined for the overall mutant PS I system yielding E_mut_ for each mutant case affording direct comparison to the WT PsaA and PsaB.

## Results

### Physiological characterization of mutant strains

The mutant strains showed differences in their color, which might be due to the different chlorophyll levels. To quantify this observation, we measured cellular chlorophyll content of the RWT and mutant cells (Table 1). Cells of the H651C/L652M, F649C/G650I and F647C mutants contained much less chlorophyll than that of the RWT strain, whereas the W643C/A644I W645C and S641C/V642I mutations did not affect their chlorophyll content significantly. To assess effects of mutations on growth, the mutant strains were transferred initially to BG-11 plates without glucose. The H651C/L652M, F649C/G650I and F647C mutants could not form visible colonies on BG-11 plates without glucose. In contrast, the mutants with W645C, W643C/A644I and S641C/V642I substitutions did not require glucose to form colonies on plates, indicating their ability of photoautotrophic growth. To obtain more quantitative results on photoautotrophic and photoheterotrophic growth, the RWT, PCRTΔB, and mutant strains were cultured in liquid medium under different light intensities and doubling times were determined. Under low light intensity (2–3 µmoles m^−2^ s^−1^) with glucose in the BG-11 medium, all mutant strains except the recipient strain PCRTΔB showed similar doubling time of 25 to 34 hours (Table 1). While the PCRTΔB strain is known to grow only under the light activated heterophotoautotrphic growth (LAHG) condition [44], in our experiments it did not grow most likely because of the excess reductants generated by PS II. Comparable rates of growth under photoheterotrophic conditions indicated that the mutations did not impair the ability to use glucose as an energy source. When the RWT and mutant strains were cultured with glucose, all mutant strains except the one with F649C/G650I substitution could grow under normal light intensity (40 µmoles m^−2^ s^−1^) with only small differences in their growth rates. However, their growth rates differed significantly when grown under high light intensity (160 µmoles m^−2^ s^−1^), even with glucose (Table 1). Light sensitivity of these mutant strains indicates that the mutations led to an imbalance between the activities of PS I and PS II. Disrupted electron transfer path between PS I and PS II could cause accumulation of electronic excitation energy and lead to dangerous triplet formation and consequent damage due to singlet oxygen. When the photoautotrophic growth of the strains was tested under normal and high light intensity, the mutants with H651C/L652M, F649C/G650I and F647C substitutions could not grow under photoautotrophic conditions, which is consistent with the observations of cell growth on plates. The mutant strains with W645C, W643C/A644I and S641C/V642I substitutions could grow photoautotrophically, but at reduced growth rates.

**Table 1:** Physiological characterization of the mutant cells.

Strains	Chlorophyll content (µg/OD_730_/ml)	Doubling Time (Hours)				
		With Glucose			Without Glucose	
		Low Light	Normal light	High Light	Normal Light	High Light
RWT	3.54±0.08	24.7±0.6	17.0±0.0	15.3±0.6	40.3±0.6	62.0±3.0
PCRTΔB	-	-1	-	-	-	-
H651C/L652M	1.47±0.06	27.7±0.6	-	-	-	-
F649C/G650I	-	34.0±1.0	-	-	-	-
F647C	1.50±0.06	31.7±0.6	28.3±1.5	-	-	-
W645C	3.47±0.05	27.0±0.0	17.6±0.6	17.6±0.6	42.3±1.2	64.7±4.5
W643C/A644I	3.51±0.05	25.3±0.6	20.3±0.6	26.3±0.6	47.3±3.1	96.7±6.1
S641C/V642I	3.51±0.09	25.3±0.6	17.7±0.6	15.7±0.6	41.0±2.0	61.7±4.5

1 : ‘-’ indicates that the strains died or did not grow.

### Photosynthetic activities of mutant strains

Differences in the growth of mutants could result from reduced PS I activity in the mutant cells. To investigate this possibility, we studied the impact of the mutations on photosynthetic activities of the membranes, which were estimated changes in oxygen concentration due to electron transport through PS I or PS II (Table 2).

**Table 2:** Biochemical characterization of the thylakoid membranes of the mutant strains.

Strains	PS II Activity (nmol O_2_•OD_730_^−1^•h^−1^)	PS I Activity^1^			P700 content of thylakoids
		DAD to MV (nmol O_2_•OD_730_^−1^•h^−1^)	PC to MV (nmol O_2_•OD_730_^−1^•h^−1^)	cyt *c_6_* to fd (µmol NADPH•OD_730_^−1^•h^−1^	(10^4^ P700•cell^−1^)
RWT	287.0±7.0 (100)	−405.0±9.0 (100)	−617.0±25.0 (100)	70.0±4.0 (100)	7.1
PCRTΔB	199.3±11.4 (69.4)	−16.7±4.2 (4.1)	Nd^2^	nd	nd
H651C/L652M	268.7±10.3 (93.6)	−95.3±7.0 (23.5)	−38.3±3.5 (6.2)	3.9±1.0 (5.6)	2.8
F649C/G650I	221.0±15.5 (77.0)	−23.0±4.6 (5.7)	nd	nd	nd
F647C	256.7±15.0 (89.4)	−121.0±13.5 (29.9)	0.0±0.0 (0.0)	0.0±0.0 (0.0)	2.5
W645C	283.0±6.6 (98.6)	−396.3±27.0 (97.9)	−606.0±23.6 (98.2)	67.0±8.5 (95.7)	6.8
W643C/A644I	278±8.2 (96.9)	−359.0±23.6 (88.6)	−599.3±21.5 (97.1)	65.3±9.1 (93.3)	7.3
S641C/V642I	285.3±10.7 (99.4)	−403.3±16.3 (99.6)	−608.7±23.5 (98.7)	67.3±5.1 (96.1)	6.8

1 : The activity results were normalized on equal cell basis and expressed as percentage of RWT level that is shown in parentheses. Level of P700 in DM extract was expressed as 10^4^ P700 per cell.

2 : nd- not detected (most or all subunits of PS I could not be detected by Western blotting analysis).

The PS II activities were not affected substantially by the amino acid replacements in a PS I core protein. The PS II activity was assessed via oxygen evolution in which electrons are transferred from water to *p*-benzoquinone *via* the PS II complex. On an equal cell basis, the PCRTΔB recipient and the F649C/G650I mutant strain had lower PS II activity than that in the RWT cells. For other mutants, the PS II activities ranged from ∼90% of the PS II activity of the RWT membranes.

PS I activity was monitored as the oxygen uptake in which electrons are donated by DAD and accepted finally by MV through the PS I complex. When the membranes of the recipient strain were used in the assay, oxygen uptake was −16.67 nmol O_2_/O.D._730_·h, which is considered as the background activity. Membranes of the RWT strain had an activity of −405 nmol O_2_/O.D._730_·h. The H651C/L652M, F649C/G650I and F647C mutants that contain substitutions in the interior of the B*-j* transmembrane helix had lower activities (24%, 6% and 30% of the RWT PS I activity, respectively). In contrast, the W645C, W643C/A644I and S641C/V642I mutations that are close to the exterior end of the helix did not affect the PS I mediated electron transfer activity significantly. P700 content of the thylakoids, as measured from the light-induced absorption changes at 820 nm, was consistent with the PS I mediated electron transfer activities (Table 2).

When plastocyanin, a physiological electron donor, was used in the assay, the membranes of mutants with W645C, W643C/A644I, and S641C/V642I substitutions showed similar activities as with the artificial electron donor. In contrast, the membranes of mutants with F647C substitution had near-zero activity and H651C/L652M substitution had lower activity with plastocyanin as the electron donor, even though artificial electron donor was able to support PS I-mediated electron transfer to 25–30% wild-type levels (Table 2). Therefore, these mutations influence the docking of plastocyanin and/or electron throughput from plastocyanin to P700. A similar trend was observed when the activity of PS I was measured as ferredoxin-mediated NADP^+^ photoreduction rate (Table 2). In these measurements, we used the physiological electron donor (cytochrome *c*_6_) and the physiological electron acceptor (ferredoxin) of PS I. Membranes of the H651C/L652M, F649C/G650I and F647C mutants lacked the PS I activity, whereas the W645C, W643C/A644I and S641C/V642I mutants had PS I activities approaching the wild type levels.

### Accumulation of PS I in thylakoid membranes of the mutant strains

**Figure 2:**
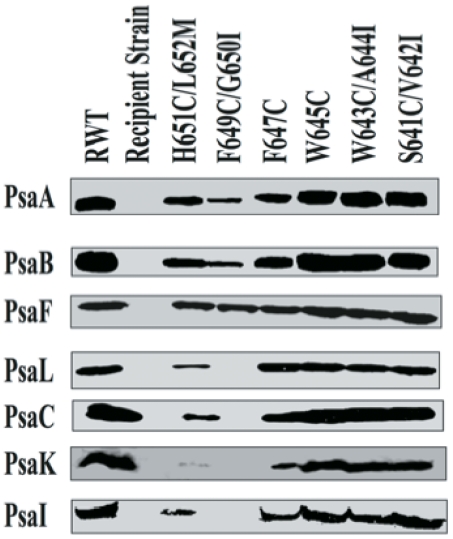
Western blotting analysis of the membranes and PS I complexes from the mutant strains. The proteins were resolved by Tricine/urea/SDS-PAGE. The antibodies against *Synechocystis* sp. PCC 6803 PsaA, PsaB, PsaC, PsaF, PsaL, PsaK and PsaI were used and the immunodetection was visualized by enhanced chemiluminescence. Membranes were isolated from the mutant strains and samples containing 5 µg chlorophyll were loaded in each lane.

Reduction in the PS I activity in the mutant membranes could result from the reduced specific activity of the complex or from the decreased levels of PS I complex. To address these possibilities, we estimated the P700 levels in the membranes (Table 2). There was a variation in the cellular levels of P700 molar ratio, ranging from undetectable levels in the F649C/G650I mutant to near RWT levels in the W645C, W643C/A644I and S641C/V642I mutants. The changes in the P700 content could result from altered properties or diminished function of the special pair of chlorophyll molecules that constitute the P700 reaction center. Alternatively, the mutations may impair assembly or reduce stability of the proteins and thus cause a reduction in the cellular levels of PS I complexes. The PsaB subunit is an integral membrane protein with eleven transmembrane helices and twelve extramembrane loops. Mutations in the PsaB subunit could affect its folding and assembly of the PS I complex. Western blotting was performed to detect levels of the PS I proteins in the thylakoid membranes (Figure 2). As expected, Western blotting showed that the PS I subunits were absent in the thylakoid membranes of the PS I-less PCRTΔB strain, whereas all subunits were detected in the RWT membranes. In the B*-j*-helix mutants, the mutants W645C, W643C/A644I and S641C/V642I contained approximately equal amounts of all seven subunits as compared to RWT. With the exception of the PsaF antibody, all other antibodies showed reduced levels of respective subunits in membranes of the mutants H651C/L652M and F647C. However, greatly reduced amounts of PsaA and PsaB subunits were detected and PsaL, PsaC, PsaK and PsaI subunits could not be detected in the membrane from the mutant F649C/G650I. Therefore, replacement of the L652, H651, F649, G650 and F647 residues reduces the level of PS I proteins in the membranes. The amount of PsaF subunit was almost the same in the membranes of all six mutants, suggesting that the levels of PsaF in the membranes are independent of the levels of assembled PS I complexes in the membranes. This conclusion is consistent with the detectable levels of PsaF in the PS I-less strains of cyanobacteria [45]. The membranes of the mutant with F649C/G650I replacement showed low levels of PsaA and PsaB proteins, but we could not detect P700 in the membranes. The P700 levels were estimated in the DM-solubilized membranes and the cellular numbers were deduced from the cellular chlorophyll ratios. The undetectable levels in the F649C/G650I mutant could have resulted from low signal to noise ratio for these membranes or destruction of P700 upon detergent solvation.

### Modification of cysteinyl residues in the mutant PS I complexes

The WT PsaA and PsaB proteins contain five cysteinyl residues that can not be modified by biotin maleimide [17], which specifically reacts with the sulfhydryl group of cysteinyl residues. Therefore, these residues are not exposed on the surface of PS I complexes. To study the role of four aromatic residues in the B*-j* helix, we changed these residues to cysteinyl residues. PS I trimers were purified and treated with biotin-maleimide. Modified PS I complexes were resolved by Tricine-urea-SDS-PAGE, probed with peroxidase-conjugated avidin, and then developed with enhanced chemiluminescence reagents. The WT PsaA and PsaB can not be modified by biotin-maleimide, however, the PsaB of mutant strains W645C, W643C/A644I and S641C/V642I could be modified (Figure S2). Therefore, small molecules at the aqueous phase may access the W645, W643 and S641 residues in the wild type PsaB. However, these results do not completely agree with the structural information. In the recently published PS I structure, W645 and W643 are located in a hydrophobic pocket, but do not point to any important cofactor [2]. S641 is accessible to the solvent in the structure of PS I.

### Electron transfer from donor proteins to the mutant PS I complexes

Results for electron transfer assays (Table 2) showed that substitutions in the H651 and F647 inhibit electron transfer from physiological electron donors to P700. Intact PS I complexes from these mutants could not be obtained in sufficient quantities to allow detailed kinetic studies. W645, W643, and S641 residues in the B-*j* helix are towards the lumenal surface of the PS I complex. These residues may interact with plastocyanin or may participate in the electron transfer. To examine this possibility, we studied electron transfer kinetics from plastocyanin to P700^+^ in the PS I complexes with W645C, W643C/A644I, and S641C/V642I replacements. To examine the activity of the mutant PS I complexes in accepting electrons, the purified PS I complexes were subjected to the photoreduction experiments using *Synechocystis* plastocyanin. Different concentrations of plastocyanin were used to determine rate constants of electron transfer (Table 3). For the RWT PS I complexes as well as for mutants, the kinetics of P700^+^ photoreduction was monophasic and the observed pseudo-first order rate constant (*k*_obs_) yielded linear dependence on plastocyanin concentration. The linear dependence of *k*_obs_ on protein concentration allowed us to calculate the second order rate constant (*k*_bim_) of P700^+^ reduction. All mutant PS I complexes reacted with plastocyanin in a similar fashion as the RWT PS I complexes, indicating that all mutant PS I complexes interact with plastocyanin according to the oriented collisional mechanism [13]. The second order rate constants for P700^+^ reduction in the mutant and the wild type PS I complexes were of the similar order of magnitude. Different concentrations of cations during the P700^+^ reduction assay did not influence the reduction kinetics in the mutants any differently than in the RWT (data not shown). When the mutant and RWT PS I complexes were treated with maleimide biotin and assayed for P700^+^ photoreduction experiments, there were no significant changes in the kinetic parameters (data not shown). Therefore, the amino acid replacements in the lumenal side of the B-*j* helix (W645C, W643C/A644I, and S641C/V642I) did not influence the electron transfer from plastocyanin to P700.

**Table 3:** Characterization of the mutant PS I complexes.

PS I complex	Chlorophyll to P700 molar ratio	K_bim_ (×10^−6^ M^−1^ s^−1^)	K_inf_ (×10^−6^ M^−1^ s^−1^)
RWT	111	8.6	9.7
W645C	121	6.3	5.9
W643C/A644I	132	5.4	6.2
S641C/V642I	103	7.2	7.7

The chlorophyll to P700 molar ratio and bimolecular rate constants for the mutant PS I complexes reduction by plastocyanin.

### Generation and selection of spontaneous revertant strains

To test the roles of histidine at the position 651 and aromatic residues at the positions 649 and 647, the mutant strains H651C/L652M, F649C/G650I and F647C were chosen to screen revertants. One revertant from F647C was isolated after screening ∼10^12^ cells. We also isolated a single revertant from ∼10^12^ cells of the F649C/G650I mutants. Revertants could not be generated from H651C/L652M after screening ∼10^12^ cells while one revertant was obtained from the H651L mutant after screening ∼10^12^ cells (unpublished results). The revertant cells could grow under high intensity light with and without glucose. They also contained similar levels of PS I complexes to that of the RWT strain. To identify the molecular nature of the reversions, we amplified the *psaA* and *psaB* genes of the revertants and determined their nucleotide sequences. The nucleotide sequencing results of the revertant from the F647C mutant showed that the TGC codon for C647 in the original mutant strain was changed to TAC codon, resulting in the substitution of the cysteinyl residue with a tyrosyl residue (Figure S3). Therefore, an aromatic residue at the position 647 is a critical structural requirement in PsaB. The nucleotide sequence of the revertant from the F649C/G650I mutant revealed that the revertant contained a substitution of the ATA codon for I650 to codon ACA for a threonyl residue (Figure S3). C649 in this double mutant remained unchanged, showing that the presence of an aromatic residue at the position 649 is not critical, but the presence of a small hydrophobic residue at the position 650 appears to be essential for the structural integrity of PsaB. The nucleotide sequence from the H651L mutant showed that the revertant contained a substitution of the CTG for L651 to a codon CAG for a glutamine residue (Figure S3).

### Steady-state spectroscopy of the revertants

**Figure 3:**
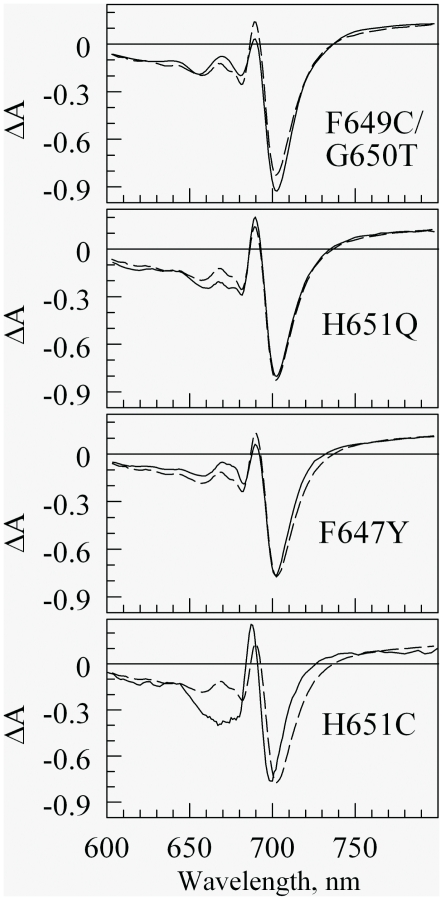
Steady-state (P700^+^ - P700) absorption difference spectra for the three revertants and the mutant of H651C. Solid and dashed curves show mutant and wild- type spectra, respectively. The y-axis shows ΔA roughly in %-level in respect to absorption in the maximum of the 680 nm band. The optimized parameters from Gaussian fit to these and other difference spectra are given in Table S2.

The traditional absorption spectra of the four studied PS I complexes coincide with the RWT and WT spectra for F647Y, F649C/G650T, and H651Q (not shown). Figure 3 shows the steady-state (P700^+^ - P700) difference spectra for F649C/G650T, H651Q, F647Y and H651C superimposed on the RWT difference spectrum. The generation of H651C mutant will be reported somewhere else. The H651C mutant is incapable of photoautotrophic growth. These are the revertants capable of photoautotrophic growth. The steady-state (P700^+^ - P700) difference spectrum for F649C/G650T is slightly different from that of the RWT. The peaks of the steady-state (P700^+^ - P700) difference spectra are ∼1 nm and ∼5 nm narrower than that of the RWT for H651Q and F647Y respectively. The largest deviation from RWT is exhibited by H651C mutant where the main photobleaching band is blue-shifted by ∼5 nm and narrowed by ∼5 nm accompanied by noticeable changes at ∼670 nm. These deviations between (P700^+^ - P700) can translate into noticeable differences in the time-resolved pump-probe kinetics for purified PS I trimers probed at 690 nm (see below). The major photobleaching components in the (P700^+^ - P700) difference spectra in Figure 3 are a broad P700 band near 700 nm, combined with a band near 655 nm that is likely dominated by differences between the Q_y_ vibronic features for oxidized and reduced reaction centers. The principal absorptive features in the difference spectrum are a sharp band (∼10 nm fwhm) at ∼690 nm, and an extremely broad band (>80 nm fwhm) centered near 800 nm. The 690 nm feature has been attributed to the monomeric Chl (hereafter C690) that emerges after oxidation of the special pair, P700^+^→C690 Chl^+^ [46]. This assignment was recently supported by Savikhin et al. [40], [42]. The 800 nm band, attributed to oxidized monomeric Chl^+^, has been used to monitor kinetics of P700^+^ in electron transfers from plastocyanins and cytochromes [21], [47]. The optimized parameters from Gaussian analyses of the RWT and all mutant difference spectra are listed in Table S2.

### Pump-probe study of energy transfer and charge separation

The pump-probe kinetics were accumulated for F647Y, F649C/F650T, H651Q and H651C mutants. The complexes were excited at 660 nm while the absorption kinetics were probed at series of wavelength between 660 and 730 nm. The obtained data for mutants and RWT was analyzed globally resulting in decay associated spectra (DAS) similarly to WT data published earlier [42]. Three of the mutants (F647Y, F649C/F650T, H651Q) and RWT could be well described with DAS with 4 decay components: ∼0.5 ps, ∼2 ps, 23 ps, >1 ns. These values are essentially the same as obtained earlier for WT and the DAS for these components mimicked closely the shape of respective spectra for WT [42]. The two shortest components, 0.5 ps and 2 ps, have been assigned earlier to energy equilibration within the main antenna pigments (0.5 ps) and energy equilibration with the redmost pigments (2 ps). The 23 ps component represents the excitation trapping time (or apparent charge separation time), while the >1 ns component arises from the formation of the long living P700^+^ state. While the DAS shapes for the H651C mutant were similar to WT, the excitation trapping lifetime was found to increase to 30 ps (rest of the lifetimes did not change). This can be readily explained by somewhat narrower and blue-shifted absorption spectrum of P700 - longer lifetimes are expected as the energy transfer from the redmost antenna pigments to the bluer P700 in mutant should be slower. Indeed, Monte Carlo simulations based on a simple pigment-to-pigment excitation hopping model that included all PS I pigments [40] showed a similar increase in excitation trapping time from 23 ps to 30 ps when the special pair absorption was blue-shifted from its original position at 699.5 nm (WT) to 695.6 nm (mutant) and the band width was narrowed from 30 nm to 25 nm.

### Theoretical calculation of energy change caused by the mutations

We calculated the energies of the WT and mutant PsaA and PsaB fragments in two ways. One way was to utilize bond dipole (Table 4), and the second way was to employ atomic charges (data not shown). The calculated minimized energy differences between the mutated and WT protein fragments compared to the unminimized values have much reduced level for all the replacements of amino acid, indicating that the steric problems were alleviated somewhat on minimization. The mutant of F649C/G650I has the highest energy differences for both minimized (ΔE_mut_ = 162 kcal/mol) and unminimized (ΔE_mut_ = 3,508 kcal/mol) protein fragments. Replacement of the Gly at 650 with Ile exhibits considerable increased steric bulk while replacing the Phe at 649 with Cys appears to offer little steric difficulty, suggesting that the replacement of the Gly at 650 with Ile would require considerable relaxation to accommodate the increased bulk of the Ile side chain and may alter other steric features of the immediate environment of the decapeptide fragment.

**Table 4:** PsaA and PsaB Fragment of PS I (Mm+Bond Dipoles) (Kcal/mol).

PsaA and PsaB Fragment	ΔE_mut_ = E_mut_−E_WT_ without minimization	ΔE_mut_ = E_mut_−E_WT_ with minimization
WT	0 (133,353)	-
H651C/L652M	444 (133,797)	38 (133,391)
F649C/G650I	3508 (136,861)	162 (133,515)
F647C	453 (133,806)	45 (133,398)
W645C	429 (133,782)	25 (133,378)
W643C/A644I	562 (133,915)	17 (133,370)
S641C/V642I	512 (133,865)	23 (133,376)

The total energies of PsaA and PsaB fragment without and with minimization are shown in parentheses.

## Discussion

Amino acid residues and polypeptide backbones near P700 are critical determinants of the structure and properties of P700 and consequently decide the functional attributes. In this work, we have examined the roles of some conserved residues near P700 in structural integrity and in electron transfer to P700. Comparison of A-*j* and B-*j* helices of PS I reveals that B-*j* helix from P700 down to the lumenal side is rich in aromatic residues. Four aromatic residues F649, F647, W645 and W643 in this region are conserved and were predicted then to be critical in maintaining PS I integrity, determining the unique physicochemical properties of P700 or providing a path for electron transfer from plastocyanin or cytochrome *c*_6_ to P700^+^. To test these hypotheses, we replaced these residues along with additional residues in the helix by dissimilar residues and examined the role of these residues in maintaining structurally sound PS I complexes and in electron transfer function of PS I. Growth of the mutant strains revealed the physiological impact of mutations. Western blotting and P700 estimation were used to analyze PS I content of the cells whereas assays with artificial or native electron donors were used to determine rates of PS I mediated electron transfer.

**Figure 4:**
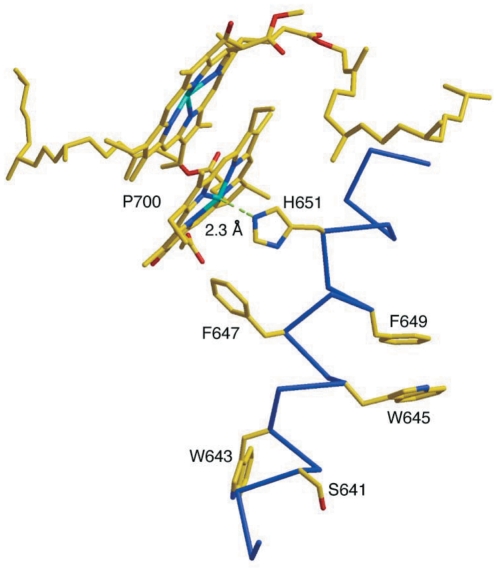
2.5 Å structure of the mutated residues in *j* helix of PsaB. The coordinates were downloaded from protein data bank (PDB ID: 1JB0) and XtalView software was used to generate the structural diagram. The nomenclature for the corresponding amino acids was changed to show the corresponding residues in *Synechocystis* sp. PCC 6803.

W645C, W643C/A644I and S641C/V642I substitutions altered regions relatively far from P700 and closer to the lumenal side. The previous investigations of P700^+^ reduction in *C. reinhardtii* suggested that PS I in this region may experience structural microrearrangements, which would allow some degree of flexibility of the PS I-plastocyanin binary complex conformation [48], [49]. The residues S641, W643 and W645 are located between P700^+^ and its electron donors plastocyanin/cytochrome *c*_6_ at the lumenal side of the protein [2]. The biotin-maleimide experiment also supported the scenario where S641, W643 and W645 can undergo conformational changes to some degree since they are predicted to be accessible to small molecules. Our functional study shows that these mutations do not influence growth of the mutant cells, PS I levels, or PS I activity when assayed using diverse techniques. Therefore, these aromatic residues are dispensable and do not play essential roles in maintaining the structural integrity of PS I complex. In addition, these residues are not essential for electron transfer from plastocyanin to P700. These results are consistent with the structural information. The rings of W645 and W643 point away from P700 (Figure 4) and do not interact with critical residues or cofactors [2], [5], [11].

Replacement of F647 with cysteinyl residue caused ∼60% reduction in PS I levels in the thylakoid membranes, indicating the importance of this residue in maintaining the integrity of PS I complex. Although significant levels of PS I were present in the membranes, these complexes were lost during purification and could not be isolated. Therefore, we believe that the reduced level of PS I in this strain is due to decreased stability of the complex and the complexes observed in the membranes are not intact. To investigate the necessity of an aromatic residue in the position of 647, the mutant F647C, which cannot grow without glucose, was transferred to plates without glucose and spontaneous revertants were selected. The true revertant of the mutant with F647C substitution contained the replacement of the cysteinyl residue at the position of 647 with a tyrosyl residue. The revertant contained normal level and activity of PS I. These results show that an aromatic residue at position 647 is critical in maintaining the integrity of PS I complex. In the 2.5 Å structure [2], the ring of F647 directly points to P700 chlorophyll pair and could contribute to the hydrophobic interactions between chlorophyll molecules and protein environment around them (Figure 4). The hydrophobic interactions seem to be precise because an additional hydroxyl group of the revertant of F647Y causes 5 nm narrower of P700^+^ - P700 spectrum. Electron transport activity of the F647C mutant membranes show that 3,6-diaminodurene (DAD), an artificial electron donor, can support electron transfer through mutant PS I complexes. DAD is a small molecule and could have an easy access to P700 for donating an electron. Plastocyanin or cytochrome *c_6_* needs to dock transiently on the PS I surface and the electron needs to travel through proteinaceous medium to P700^+^. Therefore, the mutation could disturb the docking site of plastocyanin or cytochrome *c_6_* or electron transport from plastocyanin or cytochrome *c_6_* at the PS I surface to P700^+^.

The mutant with F649C/G650I substitutions had much lower content of PS I in the thylakoid membranes, indicating that F649 and/or G650 play role in maintaining the integrity of PS I complex. To further determine whether F649 and G650 function in maintaining the integrity, spontaneous revertants with normal PS I levels were selected. The selection of revertant F649C/G650T from F649C/G650I indicates that the aromatic residue at 649 is not critical for maintaining PS I structure although a π stack is found between F649 and W645 (Figure 4). Contrastingly, a small hydrophobic residue seems to be necessary at the position 650 to maintain the integrity of PS I complex. The selection of the revertant F649C/G650T is in agreement with the structural information. The long chain of I650 in B-*j* helix could hinder sterically the neighboring B-*k* helix whereas T650 would allow packing of these two helices. The electron transport measurement showed that the mutant F649C/G650I had low PS I level, resulting in low PS I activity. The PS I activity in the mutant is not sufficient to fulfill the energy requirements for the photoautotrophic growth. The reduced PS I level may be due to the reduced stability of the complex or because of the effects on the assembly of PS I. PS I activity of the revertant F649C/G650T is very similar to RWT (data not shown). It suggests that F649 is not directly involved in the electron transport from plastocyanin to P700^+^.

The 2.5 Å X-ray structure clearly shows that H651 provides the fifth ligand to Mg^2+^ of one of chlorophyll *a* of P700 (Figure 4) [2]. Therefore, the replacement of this residue could result in changing the environment around P700 and affect its function. Inactive PS I complexes might have much higher turnover rate. Indeed, the reduced PS I level was found in the mutant H651C/L652M. Reduced PS I level and dysfunctional P700 prevent photoautotrophic growth. These results agree with the observations in the similar replacements of histidinyl residues in the reaction center of *Chlamydomonas reinhardtii* (*C. reinhardtii*) [6], [50].

The results presented here demonstrate that W645, W643 and F649 are not critical for maintaining the integrity of PS I complex and mediating the electron transport from plastocyanin to P700. In contrast, a small hydrophobic residue at 650 position and an aromatic residue at 647 position are essential for maintaining the structural integrity of PS I complex. The F647 residue may be also directly involved in the electron transfer from plastocyanin or cytochrome *c*_6_ to *C. reinhardtii* P700^+^. Its detailed function is worth to be further investigated.

It is quite interesting that in all studied revertants capable of photoautotrophic growth the (P700^+^-P700) optical spectra and kinetics of energy and charge transfer are almost identical to that in RWT. Even H651C that is incapable of photoautotrophic growth exhibits relatively efficient charge transfer function that is only marginally slower than that in RWT. We suggest that the H651C mutation may have a dramatic effect on the photostability of PS I due to the local structural change around P700. The importance of protein interactions in determining P700 properties has been examined with extensive mutagenesis in both eukaryotic algae and cynaobacteria [50]–[54]. These experiments identified the histidyl residue (656 in PsaB of *C. reinhardtii*) that interacts closely with one of the P700 chlorophylls [12]. Mutation of His656 to Asn or Ser increases the oxidation midpoint potential of P700/P700^+^ by 40 mV [12]. In a systematic mutational survey, conserved histidyl residues in the last six transmembrane segments of the PsaA and PsaB in *C. reinhardtii* were changed to glutamyl or leucyl residues by site-directed mutagenesis [50]. Double mutants with mutations in the equivalent residues in both PsaA and PsaB were screened for changes in the characteristics of P700. These studies confirmed that PsaA-His676 and PsaB-His656 in *C. reinhardtii* are the axial ligands to the P700 chlorophylls. Thus axial ligands have significant influence on the redox properties of the reaction center. However, the traditional view of the special pair as a primary electron donor has been challenged by the recent ultrafast investigation of the WT and point mutant PS I particle from *C. reinhardtii* [55]. The study provides evidence that the primary electron transfer event is initiated independently in PsaA and PsaB branches, and that initial charge separation event occurs between A_0_ and accessory chl pair and is followed by rapid reduction by P700 in the second electron transfer step. In this work, we generated mutations in the axial ligand histidyl residue as well as in the PsaB residues that are integral part of the P700-binding pocket. These mutants and revertants can be used to study further details on P700 properties.

## Supporting Information

Figure S1 **Sites of mutations in the C-terminal region of PsaA and PsaB.** Comparison of the PsaA and PsaB sequences shows transmembrane helices with gradated filling, with the darker side towards the stromal surface of the membrane. Bars above and below the sequence show stromal and luminal extramembrane loops, respectively. Residues forming two surface helices are boxed with uniform filling. The histidyl, asparaginyl or methionyl residues that are proposed to bind proximate Chl are indicated by hexagons. The binding sites for different PS I cofactors are shown.
(TIF)

Figure S2 **Modification of the mutant PS I complexes.** Purified PS I complexes containing 5 µg chlorophyll were treated with biotin-maleimide and analyzed by Tricine/urea/SDS-PAGE. The blot was probed with peroxidase-conjugated avidin and visualized by enhanced chemiluminescence reagents.
(TIF)

Figure S3 **Nucleotide sequences around the mutated sites in the mutant and revertant strains.** The PCR fragments containing the mutation sites were sequenced.
(TIF)

Table S1 **The oligonucliotides used for generating the B-j-helix mutants.**
(DOC)

Table S2 **The Gaussian parameters for 5-component fits to (P700^+^ - P700) absorption difference spectra of PS I mutants^a^.** ^a^ For each entry, the band position (in nm) is followed by its fwhm (in nm) in parentheses; the signed number gives its amplitude.
(DOC)
