# Supplementary figures and images for: Mutational Analysis of Photosystem I of Synechocystis sp. PCC 6803: The Role of Four Conserved Aromatic Residues in the j-helix of PsaB
Source: PLoS One. 2011 Sep 12;6(9):e24625. doi: 10.1371/journal.pone.0024625 (PMC3171458; doi:10.1371/journal.pone.0024625)

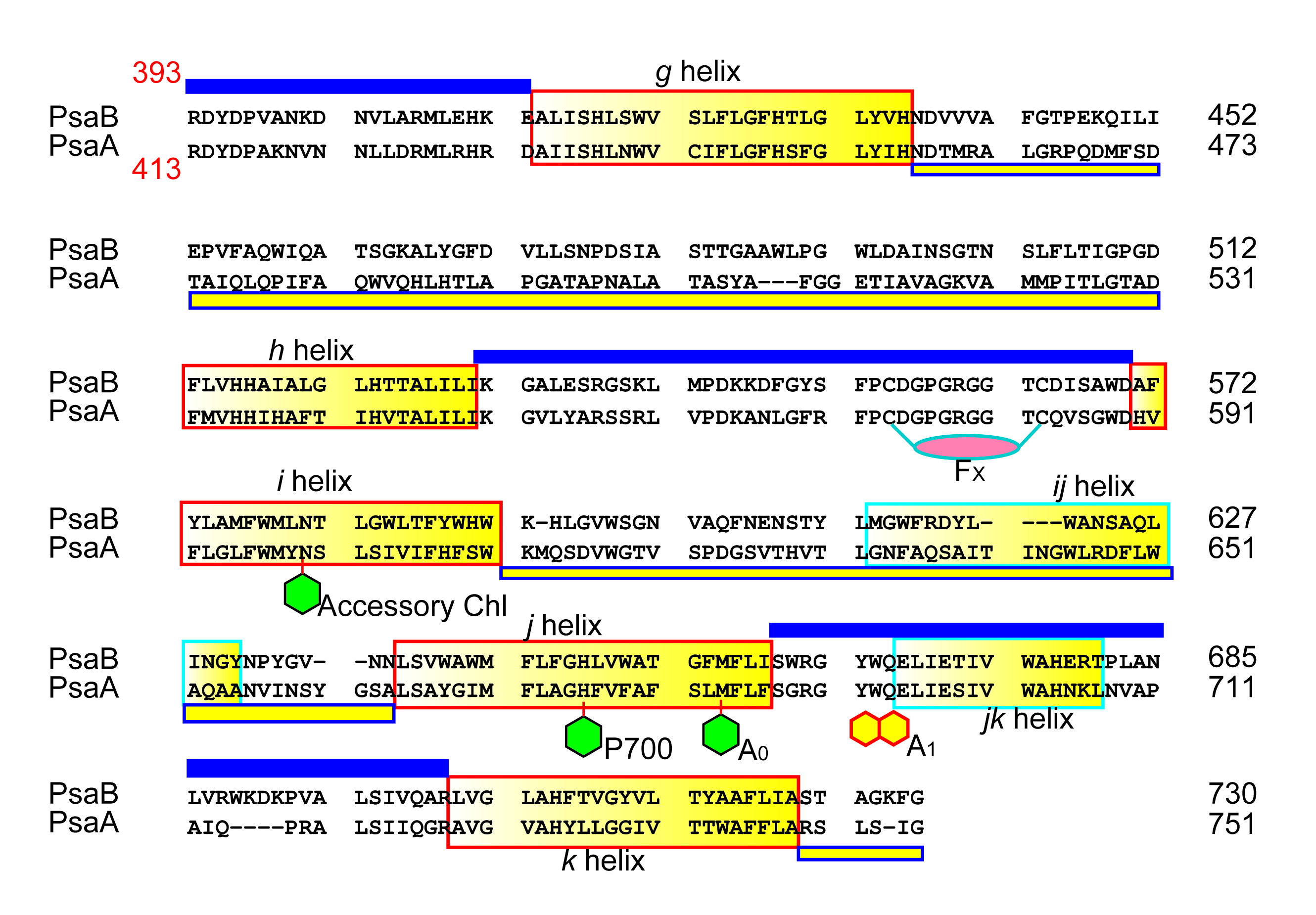

Supplement: Figure S1 — Sites of mutations in the C-terminal region of PsaA and PsaB. Comparison of the PsaA and PsaB sequences shows transmembrane helices with gradated filling, with the darker side towards the stromal surface of the membrane. Bars above and below the sequence show stromal and luminal extramembrane loops, respectively. Residues forming two surface helices are boxed with uniform filling. The histidyl, asparaginyl or methionyl residues that are proposed to bind proximate Chl are indicated by hexagons. The binding sites for different PS I cofactors are shown. (TIF) [file pone.0024625.s001.tif]

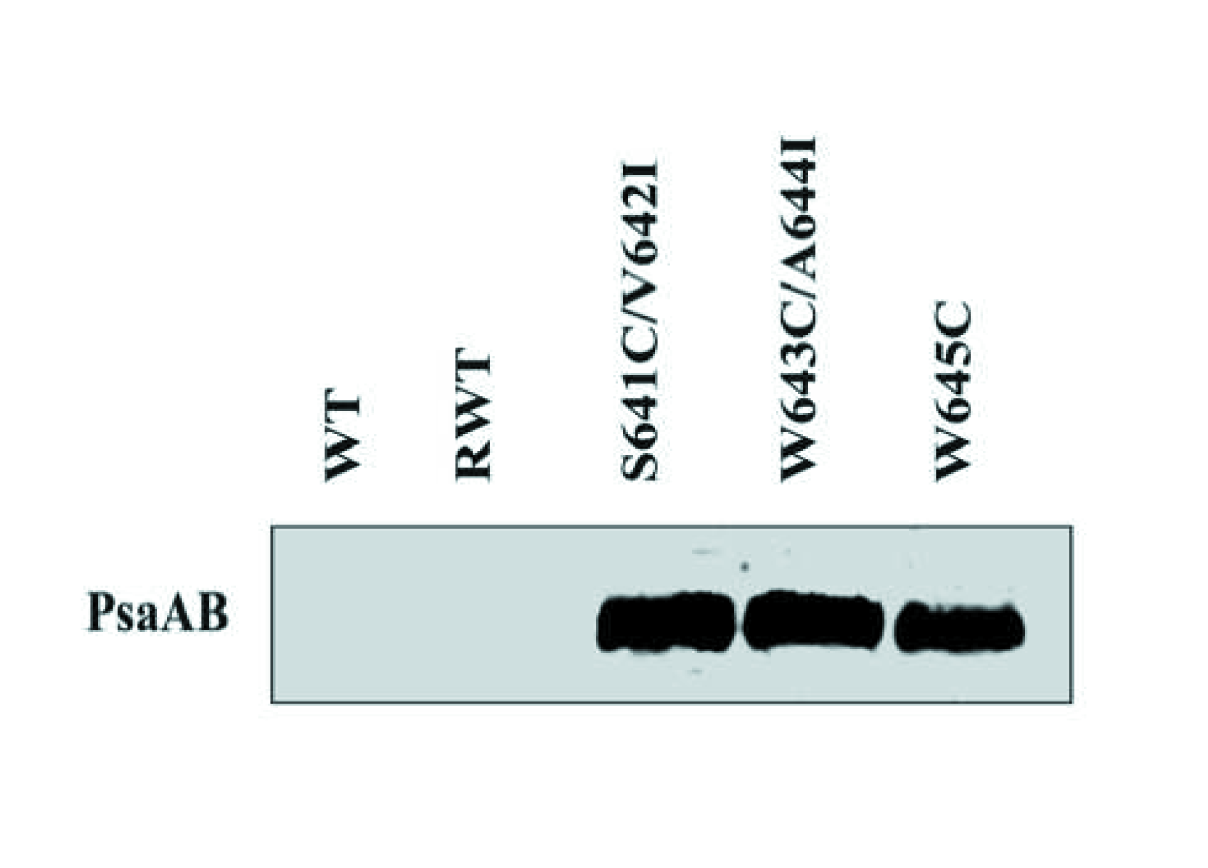

Supplement: Figure S2 — Modification of the mutant PS I complexes. Purified PS I complexes containing 5 µg chlorophyll were treated with biotin-maleimide and analyzed by Tricine/urea/SDS-PAGE. The blot was probed with peroxidase-conjugated avidin and visualized by enhanced chemiluminescence reagents. (TIF) [file pone.0024625.s002.tif]

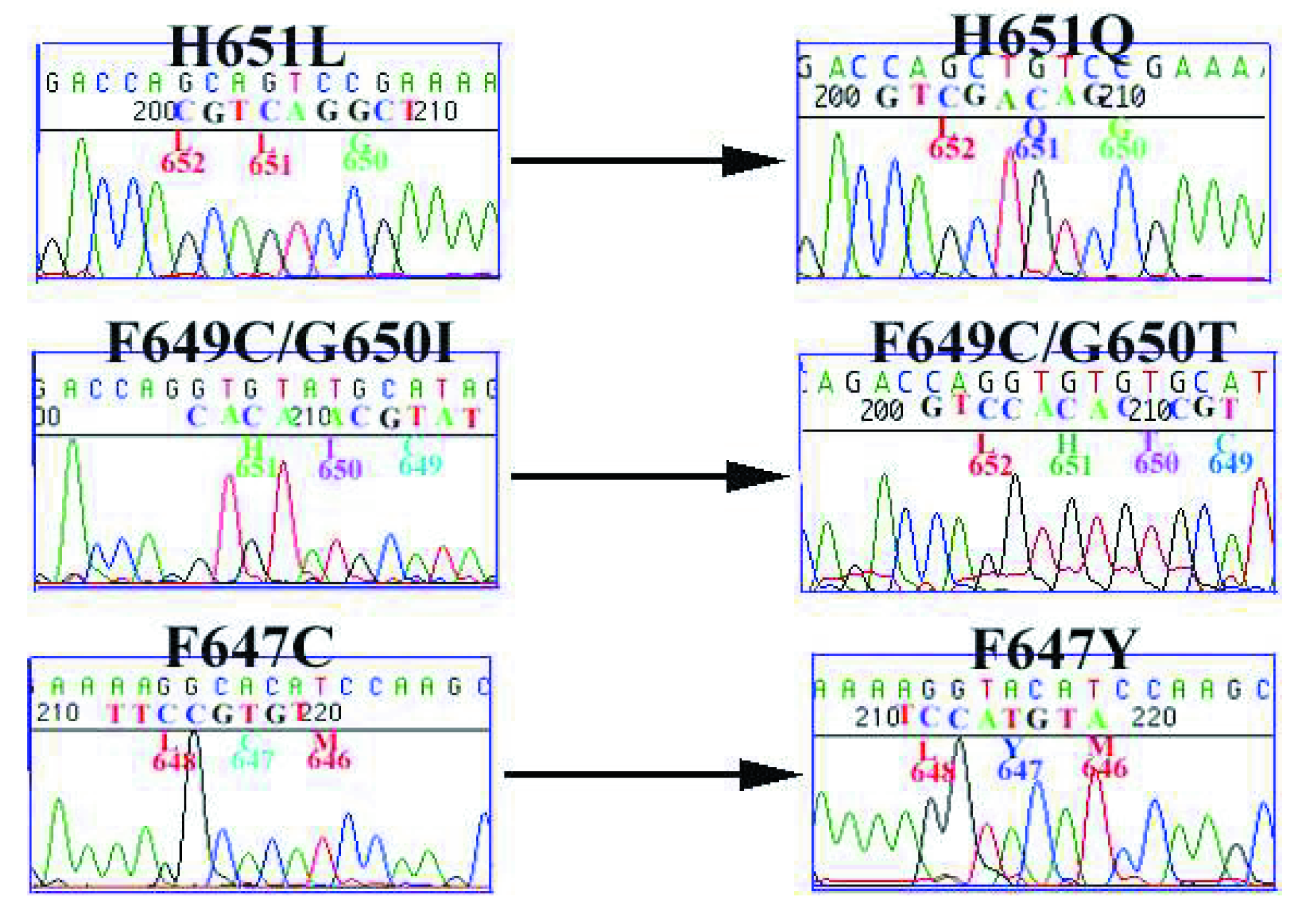

Supplement: Figure S3 — Nucleotide sequences around the mutated sites in the mutant and revertant strains. The PCR fragments containing the mutation sites were sequenced. (TIF) [file pone.0024625.s003.tif]
