# Supplementary material for: Mutational Analysis of Photosystem I of Synechocystis sp. PCC 6803: The Role of Four Conserved Aromatic Residues in the j-helix of PsaB
Source: PLoS One. 2011 Sep 12;6(9):e24625. doi: 10.1371/journal.pone.0024625 (PMC3171458; doi:10.1371/journal.pone.0024625)
Supplement: Table S1 — The oligonucliotides used for generating the B-j-helix mutants. (DOC) [file pone.0024625.s004.doc]

*Table S1. The oligonucliotides used for generating the B*-j-helix mutants

| Mutations | Oligonucleotides |
| --- | --- |
| H651C/L652M | 5’-AGCCCAGACCATGCATCCGAAAAGGAAC-3’ |
| F649C/G650I | 5’-GACCAGGTGTATGCATAGGAACATCCAAGC-3’ |
| F647C | 5’-GTCCGAAAAGGCACATCCAAGCC-3’ |
| W645C | 5’-AAAGGAACATGCATGCCCAAACTGACAG-3’ |
| W643C/A644I | 5’-GGAACATCCAAATGCATACTGACAGATTG-3’ |
| S641C/V642I | 5’-CCAAGCCCAAATGCATAGATTGTTGACACC-3’ |

*Supplementary Table II. The Gaussian parameters for 5-component fits to (P700^+^ - P700) absorption difference spectra of PS I mutants ^a^*

| Mutant | P700, 0-0 | C690, 0-0 | P700^+^, 0-0 | Vibronic | Vibronic |
| --- | --- | --- | --- | --- | --- |
| WT | 699.5 (29.4)  -0.763 | 691.2 (10.7)  +0.731 | 800 (82)  +0.111 | 633.2 (60.4)  -0.110 | 657.8 (17.7)  -0.099 |
| F647Y | 699.2 (25.2)  -0.818 | 691.6 (10.2)  +0.674 | 800 (90)  +0.111 | 656.4 (19.3)  -0.146 | - |
| F649C/  G650T | 699.8 (27.9)  -0.978 | 690.9 (11.1)  +0.743 | 800 (84)  +0.141 | 628.1 (54.1)  -0.110 | 656.3 (15.9)  -0.137 |
| H651Q | 698.6 (28.6)  -0.871 | 690.5 (10.8)  +0.993 | 800 (80)  +0.163 | 647.1 (162)  -0.307 | 659.6 (17.3)  -0.116 |

*^a^* For each entry, the band position (in nm) is followed by its fwhm (in nm) in parentheses; the signed number gives its amplitude.
