# Supplementary material for: Mutational Analysis of Photosystem I of Synechocystis sp. PCC 6803: The Role of Four Conserved Aromatic Residues in the j-helix of PsaB
Source: PLoS One. 2011 Sep 12;6(9):e24625. doi: 10.1371/journal.pone.0024625 (PMC3171458; doi:10.1371/journal.pone.0024625)
Supplement: Table S2 — The Gaussian parameters for 5-component fits to (P700+ - P700) absorption difference spectra of PS I mutantsa. a For each entry, the band position (in nm) is followed by its fwhm (in nm) in parentheses; the signed number gives its amplitude. (DOC) [file pone.0024625.s005.doc]

*Table S2. The Gaussian parameters for 5-component fits to (P700^+^ - P700) absorption difference spectra of PS I mutants ^a^*

| Mutant | P700, 0-0 | C690, 0-0 | P700^+^, 0-0 | Vibronic | Vibronic |
| --- | --- | --- | --- | --- | --- |
| WT | 699.5 (29.4) -0.763 | 691.2 (10.7) +0.731 | 800 (82) +0.111 | 633.2 (60.4) -0.110 | 657.8 (17.7) -0.099 |
| F647Y | 699.2 (25.2) -0.818 | 691.6 (10.2) +0.674 | 800 (90) +0.111 | 656.4 (19.3) -0.146 | - |
| F649C/G650T | 699.8 (27.9) -0.978 | 690.9 (11.1) +0.743 | 800 (84) +0.141 | 628.1 (54.1) -0.110 | 656.3 (15.9) -0.137 |
| H651Q | 698.6 (28.6) -0.871 | 690.5 (10.8) +0.993 | 800 (80) +0.163 | 647.1 (162) -0.307 | 659.6 (17.3) -0.116 |

*^a^* For each entry, the band position (in nm) is followed by its fwhm (in nm) in parentheses; the signed number gives its amplitude.
